# A reliable benchmark of the last 640,000 years millennial climate variability

**DOI:** 10.1038/s41598-023-49115-z

**Published:** 2023-12-21

**Authors:** Denis-Didier Rousseau, Witold Bagniewski, Hai Cheng

**Affiliations:** 1 Géosciences Montpellier, Université Montpellier, 34095 Montpellier, France; 2 Division of Geochronology and Environmental Isotopes, Institute of Physics-CSE, Silesian University of Technology, 44-100 Gliwice, Poland; 3 Lamont Doherty Earth Observatory, Columbia University, Palisades, NY 10964 USA; 4 Laboratoire de Météorologie Dynamique, École Normale Supérieure –Paris Sciences et Lettres University, 75005 Paris, France; 5 Institute of Global Environmental Change, Xi’an Jiaotong University, Xi’an, 710049 China

**Keywords:** Climate sciences, Palaeoclimate

## Abstract

How often have past climates undergone abrupt transitions? While our understanding of millennial variability during the past 130,000 years is well established, with precise dates available, such information on previous climate cycles is limited. To address this question, we identified 196 abrupt transitions in the δ^18^O record of the well-dated Chinese composite speleothem for the last 640,000 years. These results correspond to abrupt changes in the strength of the East Asian Monsoon, which align with the Greenland stadials and interstadials observed in the North Atlantic region during the last 130,000 years before present. These precise dates of past abrupt climate changes constitute a reliable and necessary benchmark for Earth System models used to study future climate scenarios.

## Introduction

Abrupt transitions in the Earth System have become a major concern in recent decades, due to increasing evidence provided by paleoclimatic records^1^. These transitions can be classified into different categories, with Tipping Points (TPs) representing ultimate jumps from one climate state to another, with no possibility of returning to the initial conditions. In recent years, TPs have been studied with growing interest due to the impact of anthropogenic activities on the various components of the Earth system and their potential future evolution, leading to conditions that have not been observed over the last 2.6 million years. Recent analyses of current climate components, under different greenhouse gas emission scenarios, suggest that the Earth may already be approaching some of these tipping points, and that others are expected over the current century^2^. However, these projections are based solely on very recent data, dating back only a few hundred years.

The Earth system is considered to be reacting relatively abruptly to current anthropogenic forcing. However, the notion of abruptness remains ambiguous, as it refers to a timescale that is difficult to assess properly. Aware of this problem, the tipping elements listed by Lenton et al.^3^, which correspond to particular domains of the Earth system, are based on long-term observations under controlled conditions that make it possible to identify the associated tipping points. For example, there is evidence that if the rate of deforestation due to forest fires and climate change is not reduced, the Amazon rainforest will soon reach a tipping point towards a savanna state^4^. This would have a direct or indirect impact on regional and global climate systems, as well as on various other ecosystems^5^. Lenton et al.^3^ have identified tipping elements that are closely linked to current climate change and therefore directly or indirectly related to anthropogenic forcing. However, interpretations of current abrupt climate changes must always refer to studies of abrupt climatic transitions evidenced in paleoclimate proxy records covering longer timescales with little or no human impact. The study of abrupt climate changes in the past has therefore become a new field of research in recent years^1^, exploring fluctuations that occurred in relatively short time intervals of several tens or, at most, hundreds of years, according to high-resolution records from the Greenland ice cores^6–8^. However, there is evidence that abrupt transitions can also be identified in the deeper time from lower resolution records, which still reveal changes or transitions that have had a considerable impact on the global dynamics of the Earth system^9–11^.

## Abrupt transitions in past climates

Recent studies of past climate changes have underlined the importance of studying abrupt transitions in the past, while recognizing the challenges associated with data quality, including timescale accuracy and quantification of associated uncertainties^12^. Studying past abrupt transitions and the mechanisms involved requires the best possible data quality. However, this can be a serious limitation when considering the sparse spatial coverage of high-resolution paleo-records, for which precise dating is essential, and the corresponding errors are often challenging to control. Nevertheless, paleoclimate records are essential for identifying tipping points in the Earth’s past and for properly understanding the underlying bifurcation mechanisms of the climate system^13^. Due to the variable quality, resolution, and dating methods of these records, careful selection is necessary to ensure the best representation of past climates^14^.

Studies of past abrupt transitions have mainly focused on ice-core records of the last climate cycle, particularly the δ^18^O records, as they offer the best possible temporal resolution. Early evidence of abrupt transitions in the δ^18^O record of Camp Century and Dye 3 Greenland ice cores^15^ was reinforced by the detection of additional abrupt transitions in the Greenland ice core NGRIP, leading to the identification of sub-events^7^. Speleothems can, however, be considered equivalent to ice cores in terms of the quality of the preserved climate signal, mainly δ^18^O variations^16–22^. When top-down layer-counting is no longer reliable, ice cores are dated using numerous independent markers, including volcanic eruptions^23^, ice flow models^24,25^, and wiggle matching with the orbital time scale^26^. In contrast, speleothems are accurately dated over possibly several climate cycles using radiometric methods with very low error bars, making them excellent archives of past climate conditions^16,27–29^.

IPCC AR6 W1 recently indicated that “abrupt responses and tipping points of the climate system, such as strongly increased Antarctic ice-sheet melt and forest dieback, cannot be ruled out (high confidence)”^30^. The same report indicates that “For global climate indicators, evidence for abrupt change is limited…” Models that exhibit such tipping points are characterized by abrupt changes once the threshold is crossed, and even a return to pre-threshold surface temperatures or to atmospheric carbon dioxide concentrations does not guarantee that the tipping elements return to their pre-threshold state. Monitoring and early warning systems are being put into place to observe tipping elements in the climate system. For global climate indicators, evidence for abrupt change is limited”. (Ref.^31^ Box TS.9, p. 109). It is clear that the main concerns of the climate community pertain to the potential irreversible thresholds that could be reached as a result of the global warming trend, which will have major repercussions on society. However, Earth System models that aim to predict future tipping points must also be able to reconstruct past abrupt changes that have led to colder climate conditions. Unfortunately, cooling events are often given less attention or significance, despite being an important component of the climate system.

## The abrupt climate transitions of the past 640 kyrs

To assess abrupt climate transitions of the past, well-dated, high-resolution records and robust statistical approaches are needed. Greenland ice cores provide such records, capturing abrupt transitions between cold and warm conditions known as Greenland Interstadials (GI) or Dangaard–Oeschger (DO) events, with a 20-year resolution that enables precise dating of these transitions^7^. Dates have also been proposed for transitions to colder conditions, known as Greenland Stadials (GS). However, the timescale of these abrupt transitions is limited to the last climate cycle, which hinders the evaluation of Earth System models over a longer time interval. To overcome this limitation, Greenland δ^18^O variations were reconstructed from EPICA δ^18^O measurements using the thermal bipolar seesaw model of Stocker and Johnsen^32^. This method enabled the detection of abrupt, GI-like warmings over the past 800,000 years with a time resolution of 50 years^33^. However, the reconstructed abrupt warming events do not follow the decadal timescale identified in Greenland for the last climate cycle. In-depth testing of Earth System models may therefore require even finer resolution records. Another high-resolution record that can be used for this purpose is the δ^18^O record of the Chinese composite speleothem (CS), which covers seven climate cycles over the past 640,000 years^34^. Its high resolution and precise dating make it one of the best paleoclimate records for detecting abrupt transitions over several climate cycles and providing a reliable benchmark for the study of past abrupt climate changes.

As paleoclimate records differ in origin, duration, and periodicity, it is essential to have an objective, automated methodology for identifying and comparing abrupt transitions. Among several methods used to detect such transitions, a new approach based on the nonparametric Kolmogorov–Smirnov (KS) test (e.g.^35^) has recently been developed and has shown promising results^36^. The KS test is applied to compare two samples drawn from a time series, one before and one after a potential jump. The KS statistic measures the difference between the empirical distribution functions of the two samples, enabling discontinuities in the time series to be identified. To refine the results and pinpoint significant transitions, the KS test is augmented by additional criteria, including a variable window size and a minimum rate-of-change threshold. Window size is a critical factor affecting the identification of transitions (see^14^ for more details). In addition to the KS test, long-term trends in maxima and minima can also be used to establish key transitions, such as Stadial–Interstadial boundaries in the Greenland ice cores. KS method’s parameters are optimized using receiver operating characteristic (ROC) analysis. Using this accurate and robust approach, it can be noted that transitions in paleoclimate records are often not correctly identified in the literature, possibly due to variable data quality and dating methods. Although the KS test is sharper and can find precise transition dates, recurrence quantification analysis (RQA) is better suited to identifying particularly important transitions that correspond to changes in dynamics. To quantitatively assess these major transitions, recognized as distinctive patterns in Recurrence Plots (RP), we employ an RQA measure known as Recurrence Rate (RR). The minima of RR, identified through prominence analysis^36^ and marked by pink crosses in Fig. 3, signify substantial shifts in the system and are thus of particular interest to us.

**Figure 1:**
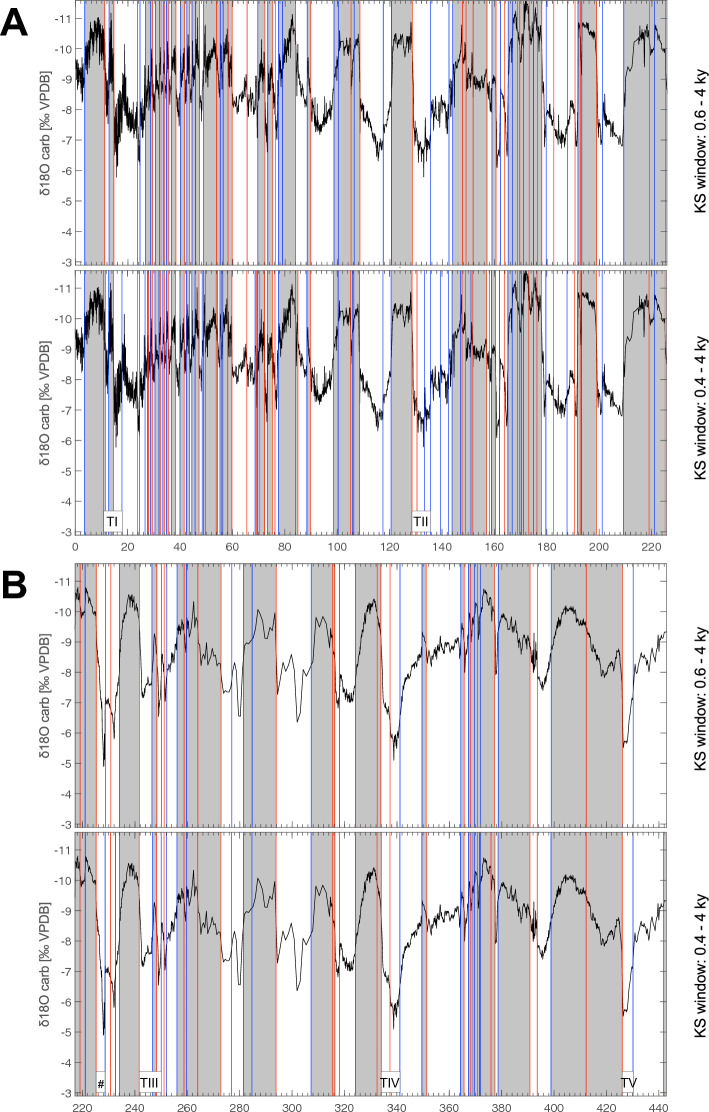

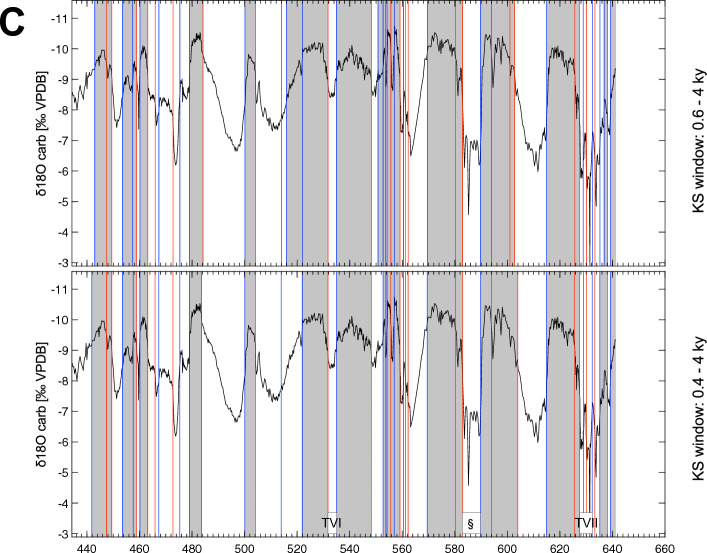
Detection of the abrupt transitions towards weak and strong East Asian Monsoon intervals. δ^18^O variations during (**A**) the 0–220 kyrs interval; (**B**) the 220–440 kyrs interval; and (**C**) the 440–640 kyrs interval with the upper and lower panels showing transitions detected with the KS test applied with the 0.6–4 kyr and 0.4–4 kyr window size range, respectively. Blue lines mark the transitions towards weak monsoon, red lines mark the transitions towards strong monsoon. Indication of the different Terminations, T1 to TVII with mention of TIII-A (#) and TVII-A (§) (see Table 3).

**Figure 2:**
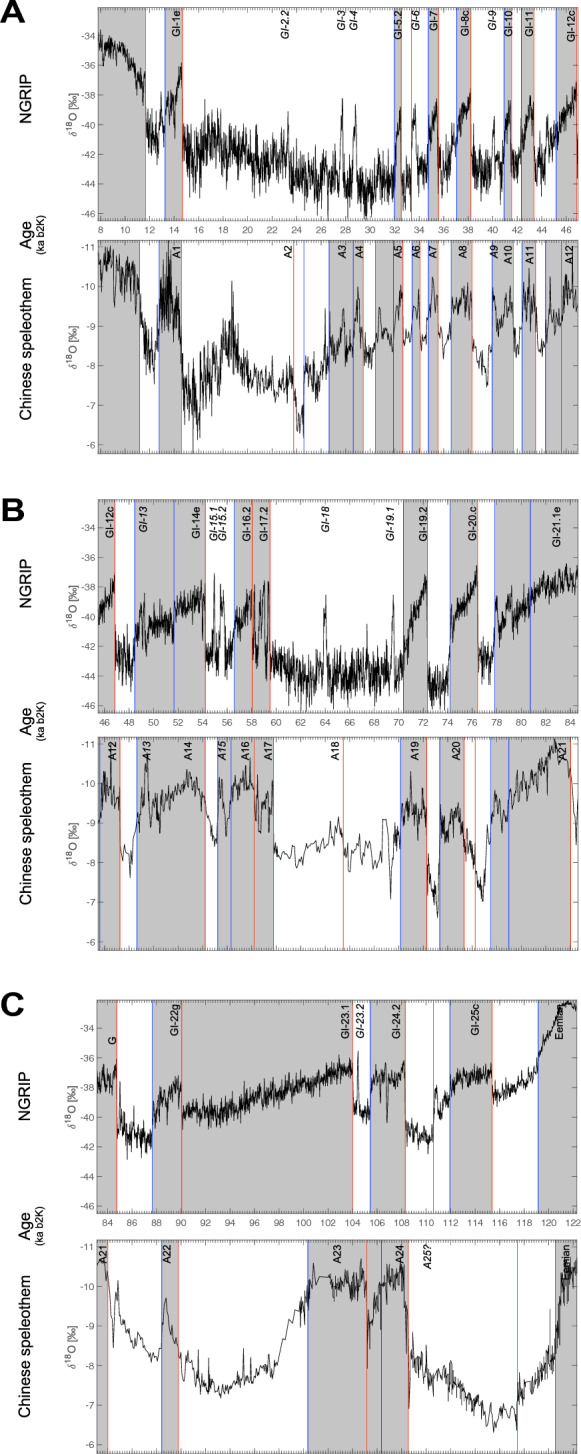
Comparison of the detected abrupt climate transitions in both NGRIP and the Chinese Speleothem δ^18^O records over the last climate cycle (see Table 2). A, during the 46–8 kyrs interval; B, the 84–46 kyrs interval; and C, the 122–84 kyrs interval. Upper panel NGRIP and lower panel Chinese Speleothem.

The use of a variable window size in the KS test can enable more accurate identification of transitions occurring over different time scales in paleoclimate records, revealing transitions that may have been missed in other analyses. In the case of the CS δ^18^O record, the KS test was applied using two different window size ranges (0.4–4 kyr and 0.6–4 kyr) to optimize transition identification (Fig. 1, Table 1). This approach detected a total of 144 similar transitions over the last 640 kyrs, as well as 27 transitions having different dates between the two analyses. While reducing the window size diminishes the statistical significance, it enables a more precise examination of the time series. As a result, the window size of 0.4–4 kyrs allowed the identification of additional 25 transitions to both moist (11) and drier (14) conditions. This improvement is not just cosmetic, as it has also enabled the detection of events found in other paleoclimate records, such as sub-events in the Greenland NGRIP δ^18^O record for the last climate cycle^7,10,36^. Specifically, the KS test of the CS δ^18^O record with the 0.4–4 kyrs window detected 58 NGRIP events and sub-events, including the GSs and GIs (Table 2). To compare detected CS transitions to strong monsoon conditions with NGRIP GIs, we followed the labeling of Chinese interstadials detected in the last 130,000 years as “A” by Cheng et al.^19^ and Wang et al.^21^. A close correspondence appears between A24 and GI-24.2 at the base and A1 and GI-1e at the top of the records (see Fig. 2, Table 2). This correspondence is also observed for older GIs found in the reconstructed Greenland δ^18^O^33^. Following the original numbering of the GIs^37^, labelled as DO events, the KS test detected 23 out of 24 of such events, with only GI-9 or A9 missing. The difficulty in identifying transitions between GI-24.2 and the base of the last climate cycle, between 135,550 years and 128,550 years BP, may be due to the complexity of the correspondence between NGRIP and CS records, as previously shown by Rousseau et al.^10^. This difficulty could also be due to the fact that these paleoclimate records from different regions may respond differently to global climate changes, leading to regional variability in the timing and magnitude of abrupt transitions.

**Table 1:** Abrupt transitions detected in the Chinese Speleothem δ^18^O composite record^34^ (All dates in ky).

China cave composite δ^18^O													
Same dates		KS window: 0.6–4 kyrs				KS window: 0.4–4 kyrs				Diff. date	Differences 0.4–0.6		
Com W	Com S	Weak monsoon	Add W	Add S	Strong monsoon	Weak monsoon	Add W	Add S	Strong monsoon		Diff weak monsoon	Diff strong monsoon	
1		3.500				3.500					0.000		
					11.129				10.824	1		− 0.306	
								1	11.514				
1		12.779				12.779					0.000		
	1				14.617				14.617			0.000	
						17.835	1						
	1				23.685				23.685			0.000	
1		24.590				24.590					0.000		
1		26.650				26.650					0.000		
						27.555	1						
								1	27.955				
		28.625				28.715				1	0.090		
	1				29.430				29.430			0.000	
1		30.420				30.420					0.000		
1		31.975				31.975					0.000		
	1				32.675				32.675			0.000	
1		33.420				33.420					0.000		
					34.065				33.995	1		− 0.070	
1		34.750				34.750					0.000		
					35.600				35.535	1		− 0.065	
		36.605				36.750				1	0.145		
	1				38.305				38.305			0.000	
		39.990				39.955				1	− 0.035		
					41.635				41.595	1		− 0.040	
1		42.475				42.475					0.000		
	1				43.615				43.615			0.000	
		44.440				44.485				1	0.045		
1		45.730				45.730					0.000		
	1				47.250				47.250			0.000	
		48.860				48.470				1	− 0.390		
						48.920	1						
								1	49.650				
					53.745				53.800	1		0.055	
								1	54.370				
1		55.230				55.230					0.000		
								1	55.745				
1		56.320				56.320					0.000		
	1				58.210				58.210			0.000	
	1				59.785				59.785			0.000	
	1				65.480				65.480			0.000	
						68.655	1						
								1	69.180				
1		69.575				69.575					0.000		
						70.155	1						
	1				72.285				72.285			0.000	
		73.350				73.400				1	0.050		
	1				75.280				75.280			0.000	
	1				76.245				76.245			0.000	
1		77.510				77.510					0.000		
1		79.035	1										
					84.030				84.130	1		0.100	
								1	85.000				
1		88.425				88.425					0.000		
	1				89.790				89.790			0.000	
1		98.650				98.650					0.000		
1		100.350				100.350					0.000		
	1				105.150				105.150			0.000	
						105.750	1						
1		106.350				106.350					0.000		
	1				108.550				108.550			0.000	
1		117.450				117.450					0.000		
1		120.550				120.550					0.000		
	1				128.550				128.550			0.000	
								1	130.450				
						133.250	1						
1		135.550				135.550					0.000		
						139.350	1						
1		142.550				142.550					0.000		
		143.950				143.850				1	− 0.100		
						147.050	1						
	1				147.650				147.650			0.000	
	1				149.050				149.050			0.000	
						151.050	1						
	1				151.750				151.750			0.000	
	1				157.050				157.050			0.000	
								1	158.150				
1		158.950				158.950					0.000		
	1				160.500				160.500			0.000	
		162.200				162.000				1	− 0.200		
	1				163.800				163.800			0.000	
1		165.150				165.150					0.000		
1		166.800				166.800					0.000		
	1				168.750				168.750			0.000	
	1				169.550				169.550			0.000	
						170.250	1						
1		171.050				171.050					0.000		
	1				173.150				173.150			0.000	
1		174.850				174.850					0.000		
	1				176.250				176.250			0.000	
	1				177.950				177.950			0.000	
1		179.650				179.650					0.000		
	1				182.550				182.550			0.000	
1		187.750				187.750					0.000		
	1				190.650				190.650			0.000	
1		191.750				191.750					0.000		
					192.650				192.750	1		0.100	
1		193.250				193.250					0.000		
					198.850				198.950	1		0.100	
1		201.150				201.150					0.000		
1		209.250				209.250					0.000		
	1				219.050				219.050			0.000	
		220.950				221.050				1	0.100		
	1				225.200				225.200			0.000	
1		228.650				228.650					0.000		
	1				230.750				230.750			0.000	
						232.650	1						
		234.000				234.150				1	0.150		
	1				241.650				241.650			0.000	
1		246.450				246.750					0.300		
					248.350				248.250	1		− 0.100	
1		250.050				250.050					0.000		
	1				251.150				251.150			0.000	
1		252.150				252.150					0.000		
1		256.050				256.050					0.000		
	1				258.750				258.750			0.000	
1		259.650				259.650					0.000		
	1				264.000				264.000			0.000	
	1				272.850				272.850			0.000	
1		276.950				276.950					0.000		
1		281.400				281.400					0.000		
1		284.700				284.700					0.000		
	1				293.900				293.900			0.000	
1		307.250				307.250					0.000		
	1				315.450				315.450				
					316.250				316.350	1		0.100	
1		318.050				318.050					0.000		
1		324.150				324.150					0.000		
	1				332.450				332.450			0.000	
					333.850				334.050	1		0.200	
	1				337.350				337.350			0.000	
1		341.150				341.150					0.000		
1		349.550				349.550					0.000		
					351.150				351.450	1		0.300	
1		364.350				364.350					0.000		
	1				365.650				365.650			0.000	
1		367.350				367.350					0.000		
	1				368.100				368.100			0.000	
						368.650	1						
1		369.550				369.550					0.000		
	1				370.750				370.750			0.000	
						371.550	1						
1		371.950				371.950					0.000		
								1	375.950				
					377.250				377.450	1		0.200	
1		378.650				378.650					0.000		
	1				390.800				390.800			0.000	
	1				393.600				393.600			0.000	
1		398.850				398.850					0.000		
	1				412.250				412.250			0.000	
	1				426.050				426.050			0.000	
1		430.150				430.150					0.000		
		442.950				441.850				1	− 1.100		
	1				447.400				447.400			0.000	
	1				449.450				449.450			0.000	
1		453.550				453.550					0.000		
1		457.350				457.600					0.250		
	1				458.900				458.900			0.000	
1		460.150				460.150					0.000		
	1				463.150				463.150			0.000	
	1				465.950				465.950			0.000	
1		467.350				467.350					0.000		
	1				472.750				472.750			0.000	
1		475.350				475.350					0.000		
1		479.050				479.050					0.000		
					484.150				483.650	1		− 0.500	
1		500.100				500.100					0.000		
	1				504.150				504.150			0.000	
1		513.950				513.950					0.000		
		515.850	1										
1		521.950				521.950					0.000		
	1				531.750				531.750			0.000	
1		535.000				535.000					0.000		
	1				548.250				548.250			0.000	
		550.750	1										
1		552.700				552.700					0.000		
	1				553.550				553.550			0.000	
1		554.200				554.200					0.000		
	1				555.650				555.650			0.000	
1		556.950				556.950					0.000		
	1				559.150				559.150			0.000	
1		560.450				560.450					0.000		
	1				561.250				561.250			0.000	
	1				562.250				562.250			0.000	
1		569.500				569.500					0.000		
	1				580.300				580.300			0.000	
	1				582.950				582.950			0.000	
1		589.800				589.800					0.000		
	1				593.900				593.900			0.000	
				1	600.950								
				1	602.700								
								1	604.000				
1		614.900				614.900					0.000		
					625.650				625.650			0.000	
					627.400				627.550	1		0.150	
		628.850				629.000				1	0.150		
	1				630.100				630.100			0.000	
1		632.300				632.300					0.000		
	1				633.300				633.300			0.000	
1		635.200				635.200					0.000		
		636.800	1										
	1				638.100				638.100			0.000	
1		639.200				639.200					0.000		
75	69		4	2			14	11		27	− 0.006	0.003	Mean
144			6				25			27	0.140	0.085	Std dev
											0.300	0.300	Max
											− 1.100	− 0.500	Min

Middle left: transitions detected with the 0.6–4 kyrs window range; middle right: transitions detected with the 0.4–4 kyrs window range. Furthest to the left: indication of common events for both window ranges. Second from the right: indication of a difference in the detected dates between the two window ranges. Furthest to the right: estimated difference in the dates between the two window ranges. *com W* common weak monsoon event, *com S* common strong monsoon event, *add W* additional weak monsoon event, *add S* additional strong monsoon event.

**Table 2:** Comparison of the abrupt transitions detected for the last climate cycle (All dates in ky).

China cave composite δ^18^O				NGRIP		CS	Greenland reconstructed δ^18^O				Comp CS-Reconst. Green.	
KS window: 0.4–4 ky			Strong monsoon	GS	GI	CI	DO pick	DO pick variable threshold	Label Barker et al.^33^	Age kyr (EDC3)	Com W	Diff CS/EDC
Weak monsoon	Com W	Com S										
3.500												
		1	10.824		Post 11.4 k e							
		1	11.514		Ante 11.4 k e		1	1	0	11.500	1	0.014
12.779	1			GS-1								
		1	14.617		GI-1e	A1	1	1	1	14.300	1	0.317
17.835	1			GS-2.1a								
		1	23.685		GI-2.2	A2			2			
24.590	1			GS-3 dust p								
26.650	1			GS-3 dust p								
27.555	1			GS3								
		1	27.955		GI-3	A3	0	1	3	27.380	1	0.575
28.715	1			GS-4								
		1	29.430		GI-4	A4	1	1	4	29.260	1	0.170
30.420	1			GS-5.1								
31.975	1			GS-5.2								
		1	32.675		GI-5.2	A5	1	1	5	32.020	1	0.655
33.420	1			GS-6								
		1	33.995		GI-6	A6	1	1	6	33.340	1	0.655
34.750	1			GS-7								
		1	35.535		GI-7c	A7	1	1	7	35.020	1	0.515
36.750	1			GS-8			1	1	?	37.780	1	
		1	38.305		GI-8e	A8	1	1	8	38.680	1	− 0.375
39.955	1			GS-9								
		1	41.595		GI-10	A10	1	1	10	41.300	1	0.295
42.475	1			GS-11								
		1	43.615		GI-11	A11	1	1	11	43.100	1	0.515
44.485	1			GS-12								
45.730	1			GI-12b?								
		1	47.250		GI-12c	A12	1	1	12	46.420	1	0.830
48.470	1			GS-13								
48.920	1			GI-13b?								
		1	49.650		GI-13c	A13			13			
		1	53.800		GI-14c?	A14	1	1	14	53.300	1	0.500
		1	54.370		GI-14e							
55.230	1			GS-15.2								
		1	55.745		GI-15.2	A15			15			
56.320	1			GS-16.1								
		1	58.210		GI-16.2	A16	1	1	16	57.080	1	1.130
		1	59.785		GI-17.2	A17	1	1	17	58.580	1	1.205
		1	65.480		GI-18?	A18	1	1	18?	63.840	1	1.640
68.655	1			GS-19.1?								
		1	69.180		GI-19.1				?			
69.575				?								
70.155	1			GS-19.2								
		1	72.285		GI-19.2	A19	1	1	19	70.680	1	1.605
73.400	1			GS-20								
		1	75.280		GI-20a?		1	1	20	74.500	1	0.780
		1	76.245		GI-20c	A20						
77.510	1			GS-21.1								
		1	84.130		GI-21.1e		0	1	21	83.180	1	0.950
		1	85.000		GI-21.2	A21						
88.425				GS-22/GI-22b								
		1	89.790		GI-22 g	A22	1	1	22	89.920	1	− 0.130
98.650	1			?								
100.350	1			?								
		1	105.150		GI-23.1	A23	0	1	23?	101.520	1	3.630
105.750	1			GS-24.1								
106.350	1			GS-24.2								
		1	108.550		GI-24.2	A24	1	1	24?	106.540	1	2.010
117.450				?			1	1				
120.550	1			GS-26								
		1	128.550		Eemian		1	1		124.220	1	4.330
	28	30	130.450						Eemian?	128.440	28	2.010

From left to right: transitions detected for the Chinese Composite Speleothem δ^18^O record with the KS test using the 0.4–4 kyr window range; similar events published for the NGRIP δ^18^O record by Rasmussen et al.^7^ with their corresponding labels; labels used by Cheng et al.^34^ for the published strong monsoon intervals; abrupt warmings reconstructed by Barker et al.^33^ using the EPICA δ^18^O record and the corresponding labels; comparison between the CS abrupt transitions detected with the KS test and the reconstructed Greenland interstadials from Barker et al.^33^. *GS* Greenland Stadial, *GI * Greenland Interstadial, *CI* Chinese label. Same conventions than in Table 1.

In the penultimate cycle (243–130 ka BP), the Chinese interstadials were labeled “B” by Wang et al.^21^. However, the KS test did not detect the most recent moist events B6 to B1 (144–131 ka BP) while identifying most of the oldest (Table 1, Extended Table 1). Comparing abrupt transitions to moister events with reconstructed GIs from the Barker et al.^33^ study, there is very little correspondence between the two records, with only 48 events, out of the 103 identified by Barker et al.^33^, having relatively close dates (see Extended Table 1). As previously mentioned, the reconstruction of Barker et al.^33^ does not reproduce the decadal time scale of the NGRIP record^7^ for the last climate cycle, leading to such result. When identifying strong monsoon events using the climate cycle boundaries defined by Lisiecki and Raymo^38^, the last two cycles have the highest number of abrupt transitions, with 21 and 28 identified for the penultimate and last cycle, respectively. In contrast, the four previous cycles, from 621 to 243 ka BP, never exceeded 11 abrupt transitions each, with 11, 9, 10, and 10 events respectively. This difference could be linked to the duration of the cycles, as only the last two cycles exceeded 110 kyrs. In addition, temporal resolution varies with climate cycles and could be considered to have a potential impact on the detection of abrupt transitions in older climate cycles. Our compilation of the composite speleothem record, using the Lisiecki and Raymo boundaries^38^ shows that this does not appear to be the case (see Extended Data Table 1).

**Figure 3:**
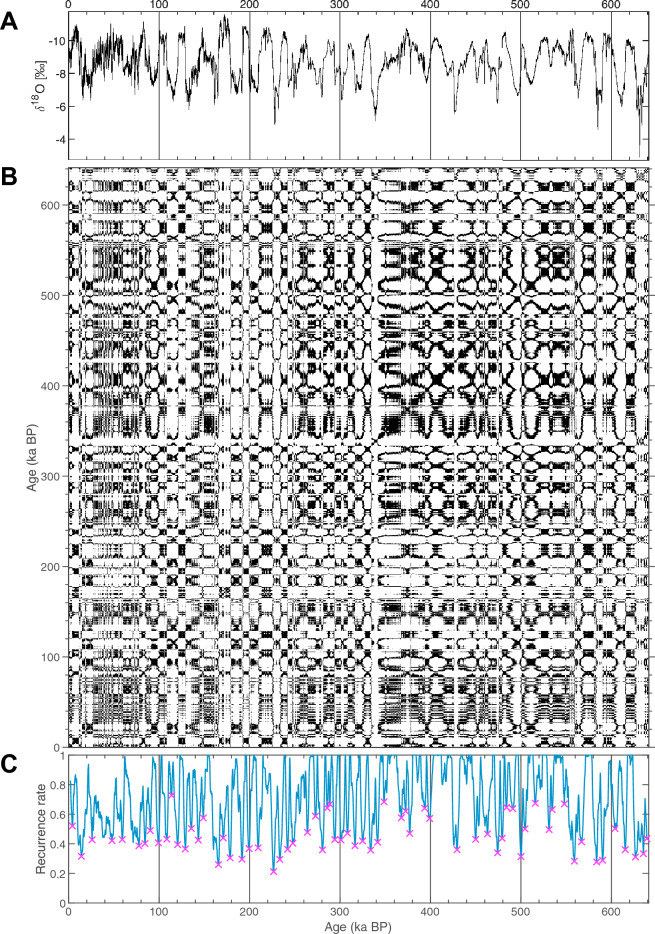
Recurrence Quantification Analysis (RQA) of the Chinese Speleothem δ^18^O composite record. (**A**) δ^18^O record over the last 640 kyrs BP, (**B**) recurrence plot (RP) of the δ^18^O time series, (**C**) recurrence rate (RR) versus time with minima marked by pink crosses selected according to their respective prominence (see Extended Table 2).

Recurrence quantification analysis carried out on the CS δ^18^O record exhibits a drift topology characteristic of a monotonic trend in variability, which corresponds to a clear dominant monsoon signature of 20 kyrs (Fig. 3). The analysis of the recurrence rates identifies 34 significant minima with a recurrence rate prominence higher than 0.5, already which correspond to transitions also identified by the KS test (Extended Data Table 2). Two main groups can be observed, separated by a main transition at 333 kyrs, with the younger transitions being more abrupt transitions than the older ones. Interestingly, the last 336 kyrs represent the time interval during which the greatest amplitude between interglacial and glacial is observed in the global δ^18^O record^38^. The analysis of the recurrence rates detects a key transition at 226.5 ka BP, corresponding to Chinese event B24, which appears to be major in terms of variability dynamics (see Fig. 3, Extended Table 2). The mechanism behind such dichotomy over the past 640 kyrs needs to be studied in greater depth using Earth system models.

The KS test is also a useful tool for determining the start and end dates of various Terminations (T) that mark abrupt transitions between glacial and interglacial stages. The precise dates of these Terminations are shown in Table 3, indicating an average duration of 6.2 kyrs ± 1.9 kyrs. However, Cheng et al.^34^ noted that these events are complex and often include one or more events corresponding to intervals of strong monsoon, surrounded by periods of weak monsoon known as “Weak Monsoon intervals” (WMI). Our analysis indicates that in the CS record, all seven identified Terminations begin with an abrupt drop in the δ^18^O signal, corresponding to the occurrence of a WMI. While five Terminations (TI, TII, TIII, TIV, TVII) show abrupt summer monsoon strengthening (high δ^18^O), characterized by either a very short or longer interstadial-like interval, two Terminations (TV and TVI) do not show such a complex structure (see Fig. 1, Table 3). This difference can be explained by the fact that these two Terminations are the shortest and correspond to low precession maxima and low insolation at 65° N (Berger^39^), compared to the other Terminations (see^34^, Fig. 1). Earth System models should nevertheless study in detail such key issue.

**Table 3:** Dates of the climate Terminations detected in the CS δ^18^O record by the KS-test.

# Termination	Base (years)	Top (years)	Duration (years)	Mean (years)	Standard dev. (years)	Max	Min
TI	17,835	10,824	7011				
TII	135,550	128,550	7000				
TIII	250,050	241,650	8400				
TIV	341,150	334,050	7100				
TV	430,150	426,150	4000				
TVI	535,000	531,750	3250				
TVII	632,300	625,650	6650	6202	1857	8400	3250
*TIII-A*	228,650	225,200	3450				
*TVII-A*	589,800	582,950	6850	5968	1877	8400	3250
MIS4/3	65,480	59,785	5695				
MIS5.2/5.1	89,790	84,130	5660	5915	1683	8400	3250

From left to right: termination number according to the marine isotope stratigraphy; start and end dates of the Terminations; duration of the Terminations, including their mean duration, standard deviation, and maximum and minimum values. Four additional intervals indicated by Cheng et al.^34^ are shown below Terminations TI–TVII.

The “2 kyr shift”, discussed by Cheng et al.^34^ and corresponding to a strong summer monsoon in East Asia during the late Holocene, was not detected by the KS-test. However, the test did identify a weak monsoon transition at 3.5 ka BP. This corresponds to a global cooling event^40,41^, which led to a significant advance of glaciers in Central Asia, the Southern Hemisphere, Northern America, Scandinavia, and the Alps^42–45^. It is also associated with tropical aridity in East Africa, South America and the Caribbean^46^, as well as with major atmospheric changes, such as the strengthening of westerlies in the North Atlantic Ocean^47^ and the increasing strength of the Siberian High^48^, which constrains the East Asian Winter Monsoon^49^.

## Implications for the analysis of past climates

What factors can be attributed to the abrupt changes detected in the composite Chinese Speleothem (CS) δ^18^O record? While the millennial variability reconstructed by Barker et al.^33^ provided possible dates for abrupt warmings during the past 800,000 years, our analysis provides precise dates of abrupt transitions from the CS δ^18^O record using a robust statistical method. These transitions are linked to East Asian summer monsoon variability, with higher δ^18^O values corresponding to weak GS-equivalent intervals, and lower δ^18^O values corresponding to strong DO events-like equivalent intervals. Furthermore, the close correspondence between abrupt transitions detected in both the CS δ^18^O and Greenland NGRIP records over the last climate cycle (last 130 kyrs) leads us to propose that all precise abrupt transitions detected by the CS serve as a benchmark for subsequent analyses of the 640 kyrs millennial variability by Earth System models to better predict future climate change scenarios.

## Materials and methods

The Chinese composite Speleothem δ^18^O record is built from stalagmites collected from four Chinese Caves: Dongge, Sanbao, Hulu and Linzhu (10.25921/xv05-8s73). It was published by Cheng et al.^34^.

Using both the augmented Kolmogorov–Smirnov (KS) test and Recurrence Quantification Analysis (RQA), our study aims to provide a comprehensive analysis of abrupt climate transitions in the composite Chinese Speleothem record. The augmented KS test is used to identify significant changes in the data, while RQA and the associated Recurrence Plot (RP) provide further insights into the recurrence patterns and system dynamics.

The first approach to identifying abrupt transitions in our datasets is to use the augmented KS test as described in Bagniewski et al.^36^. The two-sample KS test involves comparing values on either side of a value of a proxy *X* at time *t*, (*X*_*t*_), using varying window lengths. The KS statistic is calculated for all window lengths to detect abrupt transitions, with values above 0.7 considered significant. Transition detection is subsequently refined using a minimum rate-of-change threshold. The analysis begins by identifying transitions using the longest window, which has the highest sample size and therefore the greatest statistical significance. The method then integrates transitions detected using shorter windows to capture transitions on shorter time scales. To identify transitions between dominant climate modes, such as the boundaries between weak and strong monsoons in the Chinese speleothem record, we use a running window to determine the upper and lower values of the time series and identify transitions that correspond to a shift from one mode to another. Two window length ranges, 0.4–4 kyrs and 0.6–4 kyrs, were used to analyze the time series presented in this study. For more information on the KS test, see Bagniewski et al.^36^.

RPs are a powerful tool for identifying recurring patterns in time series data, including paleoclimate records. The RP for a time series takes the form of a square matrix whose two axes represent time. A dot is inscribed at a position (*i*, *j*) in the matrix when |*x*_*i*_ − *x*_*j*_|< *ε*, *ε* being the recurrence threshold. The resulting matrix provides a visual representation of recurring patterns in the time series. In addition to the visual interpretation of RP, the RQA is used to objectively quantify the recurrence patterns. Eckmann et al.^50^ distinguished between large-scale typology and small-scale texture when interpreting a square matrix of dots created by recurrence plots (RP). The most intriguing RP typology concerns recurring patterns that lack periodicity and are challenging to detect through conventional spectral analysis techniques. Marwan et al.^51^ reviewed the various methods to objectively quantify these visual RP typologies, collectively known as recurrence quantification analysis (RQA). One of the parameters derived from RQA is the Recurrence Rate (RR), which describes the probability of recurrence of system states in a given time interval. In this study, the RR is calculated using a sliding window of 4 kyrs and plotted alongside the RP. The minima of the RR (Extended Table 2), identified as abrupt transitions, are selected according to their respective prominence following the approach by Bagniewski et al.^36^.

The combination of the two methods provides a robust and comprehensive analytical capability. It has been successfully demonstrated and applied to a variety of paleoclimate records spanning the last 130,000 years to the last 66 million years from different domains (glacial, marine and continental)^9–11,14,36,52^.

### Supplementary Information

Supplementary Information 1.

## Data Availability

The CS δ^18^O composite data are available at https://www.ncei.noaa.gov/access/paleo-search/study/20450 and the codes used for KS and RQA analyses are part of the TiPES statistical toolbox available on GitHub at https://github.com/paleojump/TiPES_statistical_toolbox. The tables generated by this paper will be submitted to the PANGAEA data repository.
