# Supplementary material for: A reliable benchmark of the last 640,000 years millennial climate variability
Source: Sci Rep. 2023 Dec 21;13:22851. doi: 10.1038/s41598-023-49115-z (PMC10739820; doi:10.1038/s41598-023-49115-z)
Supplement: Supplementary file 1 — Supplementary Information 1. [file 41598_2023_49115_MOESM1_ESM.docx]

A reliable benchmark of the last 640,000 years millennial climate variability

Denis-Didier Rousseau^1^, Witold Bagniewski, Hai Cheng

Supplementary Information

Extended Data Table S1. Comparison of the abrupt transitions detected from the Chinese Speleothem and the reconstructed Greenland δ^18^O records. From left to right: Marine Isotope Stratigraphy including the Climate cycles corresponding to marine isotope stages (MIS) and their boundaries from Lisiecki and Raymo^38^; average time resolution of the speleothem record for each climate cycle; average time resolution of the for the speleothem for each climate cycle; transition dates for the speleothem record detected using the KS test with a 0.4-4 kyr window range; labels used by Cheng et al. ^34^ for the published strong monsoon intervals; dates published by Barker et al. ^33^, including the difference in dates of common events corresponding either to a strong monsoon interval in the CS record or a Greenland interstadial-like event in the reconstructed δ^18^O record. GI reconst.: Greenland Interstadial-like reconstructed by Barker et al. ^33^ (1= yes); Com CS-GISynth: Common event identified in both CS and Greenland synthetic δ^18^O records (1=yes).

| MIS | | **China cave composite δ18O** | | | **CS** | **Greenland reconstructed δ18O** | | | | |
| --- | --- | --- | --- | --- | --- | --- | --- | --- | --- | --- |
| Cycles | Boundary (kyr) | Time resolution (years) | KS window: 0.4 - 4 ky | |  |  |  |  |  |  |
|  |  |  | weak monsoon | strong monsoon | CI | Label Barker et al. (2008) | Age kyr (EDC3) | GI reconst | diff CS/EDC | Com CS-GlSynth |
|  |  |  | 3,500 |  |  |  |  |  |  |  |
| MIS 1 |  | 27 |  | 10,824 |  |  |  |  |  |  |
|  | 11.7 |  |  | 11,514 |  | 0 | 11,500 | 1 | 0,014 | 1 |
|  | 11.7 |  | 12,779 |  |  |  |  |  |  |  |
|  |  |  |  | 14,617 | A1 | 1 | 14,300 | 1 | 0,317 | 1 |
|  |  |  | 17,835 |  |  |  |  |  |  |  |
|  |  |  |  | 23,685 | A2 | 2 |  |  |  |  |
|  |  |  | 24,590 |  |  |  |  |  |  |  |
|  |  |  | 26,650 |  |  |  |  |  |  |  |
|  |  |  | 27,555 |  |  |  |  |  |  |  |
|  |  |  |  | 27,955 | A3 | 3 | 27,380 | 1 | 0,575 | 1 |
|  |  |  | 28,715 |  |  |  |  |  |  |  |
|  |  |  |  | 29,430 | A4 | 4 | 29,260 | 1 | 0,170 | 1 |
|  |  |  | 30,420 |  |  |  |  |  |  |  |
|  |  |  | 31,975 |  |  |  |  |  |  |  |
|  |  |  |  | 32,675 | A5 | 5 | 32,020 | 1 | 0,655 | 1 |
|  |  |  | 33,420 |  |  |  |  |  |  |  |
|  |  |  |  | 33,995 | A6 | 6 | 33,340 | 1 | 0,655 | 1 |
|  |  |  | 34,750 |  |  |  |  |  |  |  |
|  |  |  |  | 35,535 | A7 | 7 | 35,020 | 1 | 0,515 | 1 |
|  |  |  | 36,750 |  |  | ? | 37,780 | 1 |  |  |
|  |  |  |  | 38,305 | A8 | 8 | 38,680 | 1 | -0,375 | 1 |
|  |  |  | 39,955 |  |  |  |  |  |  |  |
|  |  |  |  | 41,595 | A10 | 10 | 41,300 | 1 | 0,295 | 1 |
|  |  |  | 42,475 |  |  |  |  |  |  |  |
|  |  |  |  | 43,615 | A11 | 11 | 43,100 | 1 | 0,515 | 1 |
|  |  |  | 44,485 |  |  |  |  |  |  |  |
|  |  |  | 45,730 |  |  |  |  |  |  |  |
|  |  |  |  | 47,250 | A12 | 12 | 46,420 | 1 | 0,830 | 1 |
|  |  |  | 48,470 |  |  |  |  |  |  |  |
|  |  |  | 48,920 |  |  |  |  |  |  |  |
|  |  |  |  | 49,650 | A13 | 13 |  |  |  |  |
|  |  |  |  | 53,800 | A14 | 14 | 53,300 | 1 | 0,500 | 1 |
| MIS 2-5 |  | 35 |  | 54,370 |  |  |  |  |  |  |
|  |  |  | 55,230 |  |  |  |  |  |  |  |
|  |  |  |  | 55,745 | A15 | 15 |  |  |  |  |
|  |  |  | 56,320 |  |  |  |  |  |  |  |
|  |  |  |  | 58,210 | A16 | 16 | 57,080 | 1 | 1,130 | 1 |
|  |  |  |  | 59,785 | A17 | 17 | 58,580 | 1 | 1,205 | 1 |
|  |  |  |  | 65,480 | A18 | 18? | 63,840 | 1 | 1,640 | 1 |
|  |  |  | 68,655 |  |  |  |  |  |  |  |
|  |  |  |  | 69,180 |  | ? |  |  |  |  |
|  |  |  | 69,575 |  |  |  |  |  |  |  |
|  |  |  | 70,155 |  |  |  |  |  |  |  |
|  |  |  |  | 72,285 | A19 | 19 | 70,680 | 1 | 1,605 | 1 |
|  |  |  | 73,400 |  |  |  |  |  |  |  |
|  |  |  |  | 75,280 |  | 20 | 74,500 | 1 | 0,780 | 1 |
|  |  |  |  | 76,245 | A20 |  |  |  |  |  |
|  |  |  | 77,510 |  |  |  |  |  |  |  |
|  |  |  |  |  |  |  |  |  |  |  |
|  |  |  |  | 84,130 |  | 21 | 83,180 | 1 | 0,950 | 1 |
|  |  |  |  | 85,000 | A21 |  |  |  |  |  |
|  |  |  | 88,425 |  |  |  |  |  |  |  |
|  |  |  |  | 89,790 | A22 | 22 | 89,920 | 1 | -0,130 | 1 |
|  |  |  | 98,650 |  |  |  |  |  |  |  |
|  |  |  | 100,350 |  |  |  |  |  |  |  |
|  |  |  |  | 105,150 | A23 | 23? | 101,520 | 1 | 3,630 | 1 |
|  |  |  | 105,750 |  |  |  |  |  |  |  |
|  |  |  | 106,350 |  |  |  |  |  |  |  |
|  |  |  |  | 108,550 | A24 | 24? | 106,540 | 1 | 2,010 | 1 |
|  |  |  | 117,450 |  |  |  |  |  |  |  |
|  |  |  | 120,550 |  |  |  |  |  |  |  |
|  |  |  |  |  |  |  | 124,220 | 1 |  |  |
|  |  |  |  |  |  | Eemian? | 128,440 | 1 |  |  |
|  | 130 |  |  | 128,550 |  |  |  |  |  |  |
|  | 130 |  |  | 130,450 | ? |  |  |  |  |  |
|  |  |  |  | 147,650 | B7 |  | 146,700 | 1 | 0,950 | 1 |
|  |  |  |  | 149,050 | B8 |  | 148,300 | 1 | 0,750 | 1 |
|  |  |  | 151,050 |  |  |  |  |  |  |  |
|  |  |  |  | 151,750 | B10 |  | 150,060 | 1 | 1,690 | 1 |
|  |  |  |  | 157,050 | B11 |  |  |  |  |  |
|  |  |  |  | 158,150 |  |  | 159,120 | 1 | -0,970 | 1 |
|  |  |  | 158,950 |  |  |  |  |  |  |  |
|  |  |  |  | 160,500 | B12 |  |  |  |  |  |
|  |  |  | 162,000 |  |  |  |  |  |  |  |
|  |  |  |  | 163,800 | B13 |  | 162,160 | 1 | 1,640 | 1 |
|  |  |  | 165,150 |  |  |  |  |  |  |  |
|  |  |  | 166,800 |  |  |  |  |  |  |  |
|  |  |  |  | 168,750 |  |  | 168,160 | 1 | 0,590 | 1 |
|  |  |  |  | 169,550 | B15 |  |  |  |  |  |
|  |  |  | 170,250 |  |  |  |  |  |  |  |
|  |  |  | 171,050 |  |  |  |  |  |  |  |
|  |  |  |  | 173,150 | B16 |  | 172,000 | 1 | 1,150 | 1 |
|  |  |  | 174,850 |  |  |  |  |  |  |  |
|  |  |  |  |  |  |  | 174,980 | 1 |  |  |
|  |  |  |  | 176,250 | B17 |  |  |  |  |  |
|  |  |  |  | 177,950 |  |  | 177,140 | 1 | 0,810 | 1 |
| MIS 6-7 |  | 72 |  |  |  |  | 178,300 | 1 |  |  |
|  |  |  | 179,650 |  |  |  |  |  |  |  |
|  |  |  |  | 182,550 |  |  |  |  |  |  |
|  |  |  | 187,750 |  |  |  |  |  |  |  |
|  |  |  |  |  |  |  | 187,840 | 1 |  |  |
|  |  |  |  | 190,650 | B19 |  |  |  |  |  |
|  |  |  | 191,750 |  |  |  |  |  |  |  |
|  |  |  |  | 192,750 | B20 |  | 192,160 | 1 | 0,590 | 1 |
|  |  |  | 193,250 |  |  |  |  |  |  |  |
|  |  |  |  | 198,950 | B21 |  |  |  |  |  |
|  |  |  | 201,150 |  |  |  |  |  |  |  |
|  |  |  |  |  |  |  | 201,420 | 1 |  |  |
|  |  |  | 209,250 |  |  |  |  |  |  |  |
|  |  |  |  |  |  |  | 215,220 | 1 |  |  |
|  |  |  |  | 219,050 | B23 |  |  |  |  |  |
|  |  |  | 221,050 |  |  |  |  |  |  |  |
|  |  |  |  | 225,200 | B24 |  | 226,200 | 1 | -1,000 | 1 |
|  |  |  | 228,650 |  |  |  |  |  |  |  |
|  |  |  |  | 230,750 | B25? |  | 229,760 | 1 | 0,990 | 1 |
|  |  |  |  |  |  |  | 232,120 | 1 |  |  |
|  |  |  | 232,650 |  |  |  |  |  |  |  |
|  |  |  | 234,150 |  |  |  |  |  |  |  |
|  | 243 |  |  | 241,650 | B26? |  | 242,180 | 1 | -0,530 | 1 |
|  | 243 |  |  |  |  |  | 243,620 | 1 |  |  |
|  |  |  | 246,750 |  |  |  |  |  |  |  |
|  |  |  |  | 248,250 | B28? |  | 248,020 | 1 | 0,230 | 1 |
|  |  |  | 250,050 |  |  |  |  |  |  |  |
|  |  |  |  | 251,150 |  |  | 251,300 | 1 | -0,150 | 1 |
|  |  |  | 252,150 |  |  |  |  |  |  |  |
|  |  |  | 256,050 |  |  |  |  |  |  |  |
|  |  |  |  |  |  |  | 256,720 | 1 |  |  |
|  |  |  |  | 258,750 |  |  | 258,480 | 1 | 0,270 | 1 |
|  |  |  | 259,650 |  |  |  |  |  |  |  |
|  |  |  |  |  |  |  | 261,160 | 1 |  |  |
|  |  |  |  | 264,000 |  |  | 263,680 | 1 | 0,320 | 1 |
|  |  |  |  | 272,850 |  |  |  |  |  |  |
|  |  |  |  |  |  |  | 276,040 | 1 |  |  |
|  |  |  | 276,950 |  |  |  |  |  |  |  |
|  |  |  |  |  |  |  | 277,900 | 1 |  |  |
| MIS 8-9 |  | 120 |  |  |  |  | 280,520 | 1 |  |  |
|  |  |  | 281,400 |  |  |  |  |  |  |  |
|  |  |  | 284,700 |  |  |  |  |  |  |  |
|  |  |  |  |  |  |  | 290,280 | 1 |  |  |
|  |  |  |  | 293,900 |  |  |  |  |  |  |
|  |  |  |  |  |  |  | 296,260 | 1 |  |  |
|  |  |  |  |  |  |  | 300,480 | 1 |  |  |
|  |  |  |  |  |  |  | 305,440 | 1 |  |  |
|  |  |  | 307,250 |  |  |  |  |  |  |  |
|  |  |  |  | 315,450 |  |  |  |  |  |  |
|  |  |  |  | 316,350 |  |  | 316,120 | 1 | 0,230 | 1 |
|  |  |  | 318,050 |  |  |  |  |  |  |  |
|  |  |  | 324,150 |  |  |  |  |  |  |  |
|  |  |  |  |  |  |  | 325,980 | 1 |  |  |
|  |  |  |  | 332,450 |  |  | 332,760 | 1 | -0,310 | 1 |
|  |  |  |  |  |  |  | 333,100 | 1 |  |  |
|  |  |  |  |  |  |  | 333,620 | 1 |  |  |
|  | 337 |  |  | 334,050 |  |  | 334,160 | 1 | -0,110 | 1 |
|  | 337 |  |  | 337,350 |  |  |  |  |  |  |
|  |  |  | 341,150 |  |  |  | 347,620 | 1 |  |  |
|  |  |  |  |  |  |  | 349,220 | 1 |  |  |
|  |  |  | 349,550 |  |  |  |  |  |  |  |
|  |  |  |  | 351,450 |  |  |  |  |  |  |
|  |  |  |  |  |  |  | 363,240 | 1 |  |  |
|  |  |  | 364,350 |  |  |  |  |  |  |  |
|  |  |  |  | 365,650 |  |  |  |  |  |  |
|  |  |  | 367,350 |  |  |  |  |  |  |  |
|  |  |  |  | 368,100 |  |  | 367,840 | 1 | 0,260 | 1 |
|  |  |  | 368,650 |  |  |  |  |  |  |  |
|  |  |  | 369,550 |  |  |  |  |  |  |  |
| MIS 10-11 |  | 80 |  | 370,750 |  |  |  |  |  |  |
|  |  |  | 371,550 |  |  |  |  |  |  |  |
|  |  |  | 371,950 |  |  |  |  |  |  |  |
|  |  |  |  |  |  |  | 372,940 | 1 |  |  |
|  |  |  |  | 375,950 |  |  |  |  |  |  |
|  |  |  |  | 377,450 |  |  |  |  |  |  |
|  |  |  | 378,650 |  |  |  |  |  |  |  |
|  |  |  |  |  |  |  | 382,000 | 1 |  |  |
|  |  |  |  |  |  |  | 386,800 | 1 |  |  |
|  |  |  |  | 390,800 |  |  | 390,960 | 1 | -0,160 | 1 |
|  |  |  |  | 393,600 |  |  |  |  |  |  |
|  |  |  | 398,850 |  |  |  |  |  |  |  |
|  | 424 |  |  | 412,250 |  |  |  |  |  |  |
|  | 424 |  |  |  |  |  | 424,900 | 1 |  |  |
|  |  |  |  | 426,050 |  |  | 426,960 | 1 | -0,910 | 1 |
|  |  |  | 430,150 |  |  |  |  |  |  |  |
|  |  |  |  |  |  |  | 435,920 | 1 |  |  |
|  |  |  |  |  |  |  | 438,820 | 1 |  |  |
|  |  |  | 441,850 |  |  |  |  |  |  |  |
|  |  |  |  |  |  |  | 443,880 | 1 |  |  |
|  |  |  |  | 447,400 |  |  | 447,540 | 1 | -0,140 | 1 |
|  |  |  |  | 449,450 |  |  |  |  |  |  |
|  |  |  |  |  |  |  | 451,940 | 1 |  |  |
|  |  |  | 453,550 |  |  |  |  |  |  |  |
|  |  |  | 457,600 |  |  |  | 454,120 | 1 |  |  |
|  |  |  |  | 458,900 |  |  |  |  |  |  |
|  |  |  | 460,150 |  |  |  |  |  |  |  |
|  |  |  |  |  |  |  | 462,460 | 1 |  |  |
|  |  |  |  | 463,150 |  |  |  |  |  |  |
| MIS 12-13 |  | 173 |  | 465,950 |  |  |  |  |  |  |
|  |  |  |  |  |  |  | 467,140 | 1 |  |  |
|  |  |  | 467,350 |  |  |  |  |  |  |  |
|  |  |  |  |  |  |  | 469,280 | 1 |  |  |
|  |  |  |  | 472,750 |  |  | 472,240 | 1 | 0,510 | 1 |
|  |  |  | 475,350 |  |  |  |  |  |  |  |
|  |  |  |  |  |  |  | 477,280 | 1 |  |  |
|  |  |  | 479,050 |  |  |  |  |  |  |  |
|  |  |  |  |  |  |  | 488,700 | 1 |  |  |
|  |  |  |  | 483,650 |  |  |  |  |  |  |
|  |  |  | 500,100 |  |  |  |  |  |  |  |
|  |  |  |  | 504,150 |  |  | 504,620 | 1 | -0,470 | 1 |
|  |  |  |  |  |  |  | 507,900 | 1 |  |  |
|  |  |  | 513,950 |  |  |  |  |  |  |  |
|  |  |  |  |  |  |  | 515,920 | 1 |  |  |
|  |  |  | 521,950 |  |  |  |  |  |  |  |
|  | 524 |  |  |  |  |  | 522,080 | 1 |  |  |
|  | 524 |  |  |  |  |  | 528,660 | 1 |  |  |
|  |  |  |  | 531,750 |  |  |  |  |  |  |
|  |  |  | 535,000 |  |  |  | 537,840 | 1 |  |  |
|  |  |  |  |  |  |  | 547,840 | 1 |  |  |
|  |  |  |  | 548,250 |  |  |  |  |  |  |
|  |  |  |  |  |  |  | 550,640 | 1 |  |  |
|  |  |  | 552,700 |  |  |  |  |  |  |  |
|  |  |  |  | 553,550 |  |  |  |  |  |  |
|  |  |  | 554,200 |  |  |  |  |  |  |  |
|  |  |  |  | 555,650 |  |  | 555,640 | 1 | 0,010 | 1 |
|  |  |  | 556,950 |  |  |  |  |  |  |  |
|  |  |  |  | 559,150 |  |  |  |  |  |  |
|  |  |  | 560,450 |  |  |  |  |  |  |  |
|  |  |  |  | 561,250 |  |  |  |  |  |  |
| MIS 14-15 |  | 238 |  | 562,250 |  |  |  |  |  |  |
|  |  |  | 569,500 |  |  |  |  |  |  |  |
|  |  |  |  |  |  |  | 579,920 | 1 |  |  |
|  |  |  |  | 580,300 |  |  |  |  |  |  |
|  |  |  |  | 582,950 |  |  |  |  |  |  |
|  |  |  |  |  |  |  | 584,920 | 1 |  |  |
|  |  |  |  |  |  |  | 586,240 | 1 |  |  |
|  |  |  | 589,800 |  |  |  |  |  |  |  |
|  |  |  |  |  |  |  | 590,380 | 1 |  |  |
|  |  |  |  | 593,900 |  |  |  |  |  |  |
|  |  |  |  |  |  |  | 595,260 | 1 |  |  |
|  |  |  |  |  |  |  | 601,920 | 1 |  |  |
|  |  |  |  | 604,000 |  |  |  |  |  |  |
|  |  |  | 614,900 |  |  |  |  |  |  |  |
|  | 621 |  |  |  |  |  | 618,600 | 1 |  |  |
|  | 621 |  |  |  |  |  | 621,020 | 1 |  |  |
|  |  |  |  | 625,650 |  |  | 625,780 | 1 | -0,130 | 1 |
|  |  |  |  | 627,550 |  |  |  |  |  |  |
|  |  |  | 629,000 |  |  |  |  |  |  |  |
|  |  |  |  | 630,100 |  |  |  |  |  |  |
|  |  |  | 632,300 |  |  |  |  |  |  |  |
| MIS 16 |  | 181 |  | 633,300 |  |  |  |  |  |  |
|  |  |  |  |  |  |  | 634,000 | 1 |  |  |
|  |  |  | 635,200 |  |  |  |  |  |  |  |
|  |  |  |  |  |  |  | 637,980 |  |  |  |
|  |  |  |  |  |  |  |  |  |  |  |
|  |  |  |  | 638,100 |  |  |  |  |  |  |
|  | 640 |  | 639,200 |  |  |  |  |  |  |  |
|  |  |  |  |  |  |  |  | 103 |  | 48 |

Extended Data Table S2. RQA of the Chinese Speleothem δ^18^O composite record ^34^. Dates of the recurrence rate (RR) minima (identified transitions), ordered according their respective RR prominence.

| Chinese Composite Speleothem δ18O | |
| --- | --- |
| RQA window: 4 ky, epsilon: 0.6 | |
| time (ka BP) | RR prominence |
| 226,533 | 0.788 |
| 165,583 | 0.741 |
| 583,683 | 0.723 |
| 559,083 | 0.717 |
| 626,683 | 0.689 |
| 500,033 | 0.686 |
| 14,083 | 0.685 |
| 191,583 | 0.679 |
| 474,233 | 0.661 |
| 333,883 | 0.643 |
| 280,633 | 0.641 |
| 429,333 | 0.640 |
| 241,983 | 0.637 |
| 128,933 | 0.635 |
| 199,533 | 0,633 |
| 209,483 | 0.626 |
| 316,583 | 0.613 |
| 120,433 | 0.606 |
| 615,383 | 0.603 |
| 77,683 | 0.602 |
| 590,283 | 0.588 |
| 324,983 | 0.581 |
| 99,283 | 0,581 |
| 247,833 | 0.575 |
| 301,083 | 0.574 |
| 294,183 | 0.571 |
| 449,983 | 0.570 |
| 377,033 | 0.530 |
| 308,233 | 0.528 |
| 108,583 | 0.525 |
| 479,283 | 0,523 |
| 463,033 | 0.520 |
| 47,883 | 0.516 |
| 531,083 | 0.504 |

References

1. Lisiecki, L. E. & Raymo, M. E. A Pliocene-Pleistocene stack of 57 globally distributed benthic delta O-18 records. *Paleoceanography* 20, PA1003, doi:10.1029/2004PA001071 (2005).

2. Cheng, H. *et al.* The Asian monsoon over the past 640,000 years and ice age terminations. *Nature* 534, 640–646 (2016).

3. Barker, S. *et al.* 800,000 Years of Abrupt Climate Variability. *Science* 334, 347–351 (2011).
